# Diet Quality Assessment in Wheelchair Users with Multiple Sclerosis

**DOI:** 10.3390/nu13124352

**Published:** 2021-12-03

**Authors:** Stephanie L. Silveira, Brenda Jeng, Gary Cutter, Robert W. Motl

**Affiliations:** 1 Department of Physical Therapy, University of Alabama at Birmingham, 3810 Ridgeway Drive, Birmingham, AL 35209, USA; bjeng@uab.edu (B.J.); robmotl@uic.edu (R.W.M.); 2 Department of Kinesiology, Health Promotion and Recreation, University of North Texas, 1155 Union Circle #310769, Denton, TX 76203, USA; 3 Department of Biostatistics, University of Alabama at Birmingham, 1665 University Blvd, Birmingham, AL 35233, USA; cutterg@uab.edu; 4 Department of Kinesiology and Nutrition, University of Illinois Chicago, 1919 W. Taylor St., Chicago, IL 60612, USA

**Keywords:** multiple sclerosis, diet, nutrition, wheelchair, dietary assessment

## Abstract

Background: Diet quality has not been distinctively examined in wheelchair users with multiple sclerosis (MS). Methods: This cross-sectional study examined the Diet History Questionnaire (DHQ) III and the Automated Self-Administered 24-h (ASA24) Dietary Assessment Tool in 128 wheelchair users with MS. Participants were prompted to complete the DHQ-III and 3 ASA24 recalls during a seven-day data collection period. Healthy Eating Index (HEI)-2015 scores were calculated for DHQ-III and ASA24, and scores were compared with normative values. Spearman’s correlation analyses (*r*_s_) estimated the associations between DHQ-III and ASA24 HEI-2015 total and component scores with supportive paired sample t-tests. Results: HEI-2015 scores for DHQ-III and ASA24 were significantly higher than normative values for total score, total protein foods, and added sugar. Correlations between HEI-2015 scores generated using ASA24 and DHQ-III were all statistically significant (range *r*_s_ = 0.23–0.69); however, significant differences between ASA24 and DHQ-III values were noted for HEI-2015 total score, total fruits, whole fruit, total vegetable, greens and beans, whole grains, seafood and plant protein, refined grains, and saturated fats. Conclusion: This study provided a novel description of diet quality in wheelchair users with MS for guiding future research promoting healthy eating in this population.

## 1. Introduction

There are an estimated one million adults in the United States living with multiple sclerosis (MS), and a vast majority of them are interested in diet for managing MS and its expression [1,2]. Diet is defined as the foods and beverages habitually consumed by organisms. The definition of a healthy diet is constantly shifting, yet it is well established that components of diet can improve metabolic and physiologic dysfunction associated with chronic health conditions [3]. Diet may be of particular interest among persons with MS as a method for management of debilitating symptoms such as cognitive impairment, mobility, disability, fatigue, bowel and bladder dysfunction, and emotional disturbances, as well as management of other comorbid health conditions [4]. To date, limited evidence is available regarding adjunctive approaches such as diet that may improve MS symptoms and slow disease progression among individuals with more severe MS, such as those who use wheelchairs for mobility.

The growing body of evidence examining diet in persons with MS has focused on specific constituents and/or supplements such as vitamin D, diet protocols low in fat, or timing of intake such as intermittent fasting [5,6,7]. For example, one study examined the effects of vitamin D supplementation over 96 weeks in 71 adults with MS [8]. The results suggested no changes in relapse, disability status, MS functional composite (MSFC) or its components, grip strength, or fatigue, despite evidence that lack of vitamin D intake is associated with the development on MS [8,9]. This body of research has expanded our understanding of diet in MS; however, many diet protocols overlook the importance of diet quality and nutrient density that may be key for improving MS symptoms [10].

There is a small body of evidence examining diet quality in MS. For example, a recent study examined diet quality among approximately 7000 adults with MS in the North American Research Committee on MS (NARCOMS) Registry [11]. Data collection included a short diet screener for creating a unique overall diet quality score based on intake of fruits, vegetables and legumes, whole grains, added sugars, and red/processed meats [11]. Results indicated that participants with worse disability (i.e., bilateral support, wheelchair users or bedridden) reported worse overall diet quality, whereas participants with higher diet quality scores reported lower symptoms of depression, pain, fatigue, and cognitive impairment [11]. That study underscored the need for further understanding of diet among persons with severe MS, such as wheelchair users. It is important to assess whether diet quality is a consequence of disability or a precursor to it, yet there are a lack of studies examining diet quality when applying various common measures of diet for assessing diet quality in MS.

The current study described diet quality based on Healthy Eating Index (HEI) scores among a sample of wheelchair users with MS. We compared two common measures of diet quality for generating HEI scores, specifically the Automated Self-Administered 24-h Recall (ASA24) and Diet History Questionnaire (DHQ) III. We selected these two measures based on the literature highlighting the validity and reliability of these tools in various populations [12]. Such inquiry provides a key step toward understanding the fundamental components for improving diet in persons with MS and guiding future research on selecting appropriate tools for measurement of the effects of diet and/or diet changes among persons with severe MS, particularly wheelchair users. This study focused on wheelchair users with MS given the stark gap in the literature regarding health behaviors among persons with severe MS and previous research highlighting worse diet quality in persons with more severe disability.

## 2. Materials and Methods

### 2.1. Study Design and Setting

Data were derived from a cross-sectional study examining health behaviors among wheelchair users with MS. Data collection occurred between February 2020 and July 2021.

### 2.2. Participants

Participants were recruited through advertisements via the National MS Society, iConquerMS, and NARCOMS. Interested participants contacted the research team directly and were screened for the following inclusion criteria: (a) age 18 years or older, (b) diagnosis of MS, (c) use of a wheelchair for mobility ≥ 50% of the day, (d) Internet access, and (e) willingness to complete the study protocol. The current study included participants who completed both measures of diet.

**Figure 1:**
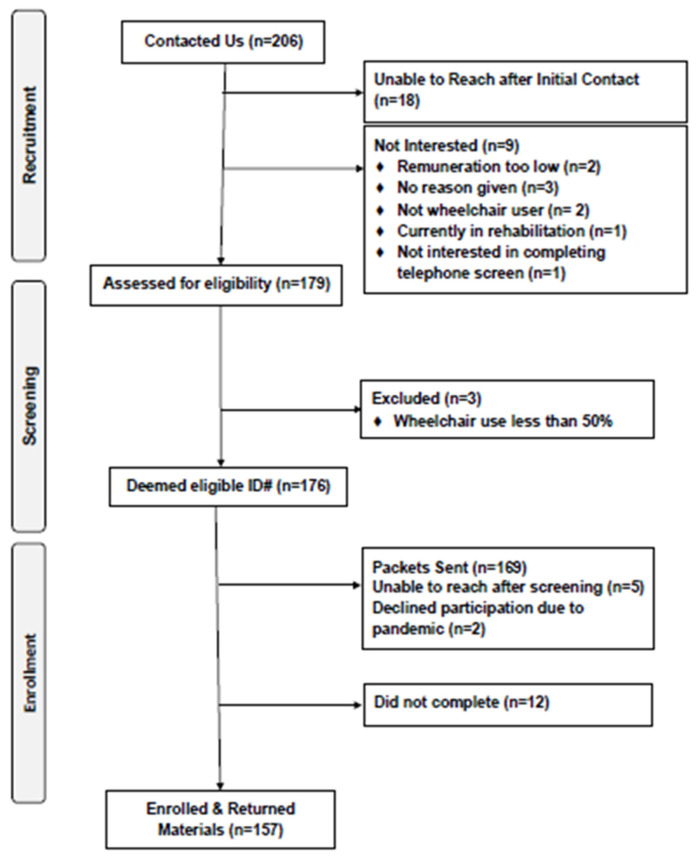
CONSORT Diagram.

We were contacted by 206 interested individuals, and 179 were formally assessed for eligibility (Figure 1). One hundred seventy-six individuals were deemed eligible initially; however, 169 packets were sent given that seven participants were lost to follow-up. Twelve individuals dropped out after receiving packets, and among the 157 packets received back, 128 were deemed eligible for analyses based on completion of both ASA24 and DHQ-III. The reasons for exclusion included: no valid ASA24 dietary recall data (*n* = 15), incomplete DHQ-III data (*n* = 10), and incomplete demographics questionnaire (*n* = 4).

### 2.3. Procedures

All study procedures were approved by the University of Alabama at Birmingham Institutional Review Board (IRB-300004359). Upon confirmation of eligibility via telephone screening, participants were invited to enroll in the study. The research team followed-up via phone to confirm it was an appropriate time to send the package in the weeks following screening (i.e., packets were sent in groups, not individually after each screening call). All procedures were undertaken remotely. Participants were mailed a packet that included two copies of the informed consent document and instructions for completing questionnaires online. Links and prompts to complete all questionnaires were delivered via e-mail as well as optional text messages if participants preferred prompts via mobile phone. Upon confirmation of receipt of the study packet, participants were prompted to complete two separate questionnaires at any time during the seven-day data collection period. Those questionnaires included (i) a battery to be completed using Qualtrics survey software, and (ii) the DHQ-III using the proprietary website. The ASA24 protocol aligned with standard practice wherein participants were prompted during the seven-day data collection period on three random, non-consecutive days to complete a 24-h dietary recall using the ASA24 website. Participants received remuneration for completing the seven-day study protocol and were only considered enrolled if they returned a signed informed consent document.

### 2.4. Measures

#### 2.4.1. Food Frequency Questionnaire

The Diet History Questionnaire (DHQ) III was utilized for assessing overall diet quality in the current study [13]. The DHQ-III is freely available for researchers, and participants were provided a unique username and password for accessing the protocol. The DHQ-III consists of 135 food and beverage line items and 26 dietary supplement questions regarding dietary intake over the previous 12-month period. Some line items included additional prompts and questions that resulted in a total assessment of 263 foods/beverages in the database. Participants were able to take breaks as needed during completion of the DHQ-III, and there were not any restrictions placed on the time frame for completing the survey as long as participants completed it during the study data collection period.

#### 2.4.2. 24-h Dietary Recall

The Automated Self-Administered 24-h (ASA24) Dietary Assessment Tool (version 2018), developed by the National Cancer Institute, was utilized for collection of 24-h dietary recalls [14]. ASA24 dietary recalls were completed online using their website, and comprehensive validation of the ASA24 software has been completed in various populations [15]. The ASA24 uses a multi-pass method wherein participants are guided through a series of steps for reporting intake of all food and drinks during the previous 24-h period. Participants were prompted on three random, non-consecutive mornings. Prompts included the website address link and their unique username and password. Prompts were structured with an overarching goal to align with best practices for dietary recalls, specifically three diet recalls during the seven-day data collection period that are non-consecutive and include two weekdays and one weekend day [15]. Dietary recalls were considered invalid if the overall intake was below 500 calories, and valid days were combined for each participant to yield an overall mean [16].

#### 2.4.3. Health Eating Index (HEI)-2015 Scoring

The HEI-2015 is a standard metric that can be used to assess conformance with the 2015–2020 Dietary Guidelines for Americans [17]. The HEI-2015 total score is based on 100 points [18]. The HEI-2015 includes 13 component scores based on common food groups or nutrients with a minimum score for each component of 0 and a maximum score of 5 or 10, depending on the component, where higher total scores reflect better diet quality. The DHQ III software automatically generates values for HEI-2015 total and the 13 component scores. Regarding ASA24 data, equivalent HEI-2015 total and 13 component scores were calculated using the SAS code provided by the National Cancer Institute using SAS, versions 9.4 (SAS Institute Inc., Cary, NC, USA). ASA24 daily means were averaged for each participant across total complete days, and participants were included if they completed at least one day.

#### 2.4.4. Demographic and Clinical Characteristics

Participants self-reported demographic characteristics including biological sex, marital status, age, employment status, race, and level of education. Participants self-reported MS clinical characteristics including MS clinical course, year of MS diagnosis, and the type of wheelchair used for mobility.

### 2.5. Data Analyses

Primary data analyses were conducted using IBM SPSS Statistics for Windows, version 27 (IBM Corp., Armonk, NY, USA). Descriptive statistics were utilized for summarizing the sample demographic and clinical characteristics, as well as HEI-2015 total and component scores generated using ASA24 and DHQ-III. Data were checked for normality using Shapiro–Wilks tests. General population HEI-2015 mean scores from the USDA website are included for descriptive comparison [19], and significant differences between those values and ASA24 and DHQ III data were assessed using one-sample t-tests. Spearman’s correlation coefficient (*r*_s_) analyses were applied for assessing the association between HEI-2015 total and component scores estimated from ASA24 and DHQ III data, and paired sampled t-tests were utilized to support correlation analyses for assessing differences in scores.

## 3. Results

### 3.1. Participants

Demographic and clinical characteristics of the 128 participants are presented in Table 1. The mean age among participants was 61 ± 10 years, and years since MS diagnosis was 23 ± 10 years. Seventy-four participants reported using a power wheelchair/scooter as the primary mobility device and 54 manual wheelchair, and 109 participants (85%) reported a progressive clinical course. The majority of participants identified as female (*n* = 95, 74%), married (*n* = 81, 63%), White (*n* = 114, 89%), not currently employed (*n* = 111, 87%), and having a college degree or more (*n* = 92, 72%).

### 3.2. Healthy Eating Index 2015 Scores

Table 2 provides HEI-2015 total and component scores for this study sample using ASA24 dietary recalls and DHQ-III compared with the mean HEI-2015 scores from all Americans (2+ years) calculated using NHANES 2015–2016 data. HEI-2015 scores for DHQ-III were significantly higher than population norms, indicating adequate or moderate intake, for total score (t (1) = 54.36, *p* = 0.01), total fruits (t (1) = 59.00, *p* = 0.01), total vegetables (t (1) = 14.2, *p* = 0.05), greens and beans (t (1) = 21.67, *p* = 0.03), dairy (t (1) = 23.00, *p* = 0.03), total protein foods (t (1) = 19.00, *p* = 0.03), sodium (t (1) = 19.50, *p* = 0.03), added sugar (t (1) = 14.60, *p* = 0.04), and saturated fats (t (1) = 52.00, *p* = 0.01). HEI-2015 scores for ASA24 were significantly higher than population norms, indicating adequate or moderate intake, for total score (t (1) = 16.65, *p* = 0.04), whole grains (t (1) = 21.00, *p* = 0.03), total protein foods (t (1) = 19.00, *p* = 0.03), seafood and plant protein (t (1) = 13.29, *p* = 0.05), and added sugar (t (1) = 13.36, *p* = 0.05). Collectively, this descriptive analysis indicates that this sample of persons with MS was significantly different from the general population mean for total score, total protein foods, and added sugar when examining both measures of diet included in the current study.

### 3.3. Bivariate Correlation between Healthy Eating Index 2015 Scores Generated Using ASA24 Dietary Recalls and the Diet History Questionnaire III

Correlations between HEI-2015 scores generated using ASA24 and DHQ-III were all statistically significant (range *r_s_* = 0.23–0.69; Table 3). Strong correlations were observed for HEI-2015 total score (*r_s_* = 0.60, *p* = 0.001) and five components: total fruits (*r_s_* = 0.69, *p* = 0.001), whole fruits (*r_s_* = 0.62, *p* = 0.001), total vegetables (*r_s_* = 0.54, *p* = 0.001), added sugar (*r_s_* = 0.51, *p* = 0.001) and dairy (*r_s_* = 0.50, *p* = 0.001). Moderate correlations were observed for six components: greens and beans (*r_s_* = 0.47, *p* = 0.001), sodium (*r_s_* = 0.46, *p* = 0.001), whole grains (*r_s_* = 0.43, *p* = 0.001), refined grains (*r_s_* = 0.38, *p* = 0.001), saturated fat (*r_s_* = 0.33, *p* = 0.001), and seafood and plant protein (*r_s_* = 0.32, *p* = 0.001). Weak correlations were observed for fatty acids (*r_s_* = 0.26, *p* = 0.004) and total protein foods (*r_s_* = 0.23, *p* = 0.009). Paired sample *t*-tests were used to assess significant differences between total score and component values, with results presented in Table 3. Significant differences between ASA24 and DHQ-III values were noted using paired sample *t*-tests for HEI-2015 total score (t (127) = 5.37, *p* = 0.001), total fruits (t (127) = 7.45, *p* = 0.001), whole fruit (t (127) = 5.96, *p* = 0.001), total vegetable (t (127) = 2.31, *p* = 0.02), greens and beans (t (127) = 2.91, *p* = 0.004), whole grains (t (127) = −2.28, *p* = 0.02), seafood and plant protein (t (127) = 4.06, *p* = 0.001), refined grains (t (127) = 3.37, *p* = 0.001), and saturated fats (t (127) = 3.95, *p* = 0.001).

## 4. Discussion

Our results indicated that wheelchair users with MS report better overall diet quality than the general population of Americans based on a comparison with normative values. The specific components of diet that exceeded the general population means when examining both measures included total score, protein foods, and added sugar. We identified areas of interest for improving diet among wheelchair users with MS, namely whole fruits, dairy, total protein foods, and seafood and plant protein, given that mean scores were below normative values. Further analyses on HEI-2015 scores generated from ASA24 and DHQ-III demonstrated significant bivariate associations; however, additional analyses indicated significant opportunities for additional inquiry regarding the measurement of diet quality in wheelchair users with MS.

Results from this study indicate high scores for HEI-2015 total scores, several adequacy component scores, and all moderation component scores among wheelchair users with MS. It is important to note these positive indicators related to intake of essential components of the diet quality such as fruits and vegetables, however there are opportunities for improvement. Increased consumption of nutrient dense, whole foods may lead to positive overall health outcomes such as reduced incidence of vascular comorbidities (i.e., diabetes, hypertension, heart disease, hypercholesterolemia, and peripheral vascular disease) that are associated with more rapid disability progression among persons with MS [20]. Concurrently, when promoting increased intake of fruits and vegetables, dysphagia (i.e., impairment in chewing and swallowing) must be considered as it is estimated to impact up to 43% of persons with MS [21,22].

Dairy consumption was low among participants compared with the general population mean; this is not surprising given common recommendations for dairy product elimination in persons with MS [23]. This underscores the need for further inquiry regarding protein intake from various sources in this population, as this may include dairy products for some individuals who can tolerate or prefer dairy. Dietary protein intake among wheelchair users with MS may be pivotal for supporting the preservation and proliferation of muscle tissue similar to established concerns and protocols in other populations that use wheelchairs as a primary mobility device, such as spinal cord injury [24]. Therefore, we assert that future research may focus on the association between protein intake and MS disease severity, particularly related to mobility and functional muscle in the upper body that is critical at this stage of MS disease, to guide dietary recommendations for persons with MS.

We located one seminal research study in a large sample of adults with MS reporting that diet quality is worse with greater disease severity based on the Patient-Determined Disease Steps [11]. Another recent cross-sectional study of 261 adults with MS examined the association between dietary patterns and disability measured using the Extended Disability Status Scale and reported no significant association [25]. Both studies utilized a Food Frequency Questionnaire with similar underlying assumptions and structure to the DHQ-III applied in the current study; however, both samples included primarily individuals with mild-to-moderate disease severity and relapsing remitting clinical course. The current study provides a novel contribution to the literature by comprehensively assessing wheelchair users with MS, who are among the subpopulation of persons with the most severe MS disease. This population is particularly unique given the limited evidence and availability of disease modifying therapies for MS disease management; therefore, engagement in positive health behaviors such as positive dietary behaviors may be key to promoting overall health and wellbeing.

One primary goal of the current study was to examine the association between two common measures of diet for assessing diet quality given the paucity of research examining the validity of self-reported diet measures in persons with MS. One recent paper estimated underreporting of 24% kcals/day in a sample of persons with MS when using the ASA24 [26]. These findings are concerning within the context of the current study, particularly for the HEI-2015 moderation component scores because numerous foods and beverages may have been missed. In the current study, strong associations were reported for HEI-2015 total and component scores when using the ASA24 and DHQ-III; however, supportive analyses indicated significant differences for 8/14 scores leaving a pressing need for further research. The aforementioned study reported that the ASA24 is challenging and burdensome for many adults with MS to complete [23]. Therefore, given the significant burden on participants (i.e., missing ASA24 data was the most prevalent reason for exclusion) and researchers in completing the ASA24 protocol, we conclude that when assessing overall diet quality, the DHQ-III may be a more suitable tool.

### Limitations

This study is not without limitations. We acknowledge the need for development of appropriate and robust tools for the assessment of diet among persons with MS given cognitive impairment, specifically working memory, likely impacts the validity of recall-based reporting methods. Participants in the current study were required to use the Internet and e-mail in order to participate and therefore may not be representative of the overall population of wheelchair users with MS; however, we note that technological proficiency was not a factor among individuals who were excluded during telephone; the only factor for exclusion during screening was wheelchair use of less than 50% of the day. Our sample size was based on funds available through a pilot study mechanism. Further, participants in this study may not be representative of subgroups within the MS population and represent a population with greater resources for healthy diet given the high education level and primarily White race. The current study was funded in January 2020 wherein nine participants completed data collection during February 2020, and the rest were following COVID-19 shut-downs and restrictions that may have impacted dietary patterns; however, these data provide a current picture of diet quality among wheelchair users with MS using both a recent 24-h recall method (ASA24) and an extended 12-month frequency method (DHQ-III) that may guide the development of future dietary interventions. General population comparison data in this study included all Americans 2+ years old; however, there is variation by age group in components of diet quality, and particularly protein intake among older adults should be examined in future studies. We highlight one strength of this study as being the opportunity to interact with persons with MS during the COVID-19 pandemic and potentially bring diet into their awareness.

## 5. Conclusions

The current study provided the first focal examination of diet in persons with MS who use wheelchairs for mobility. Wheelchair users with MS report better overall diet quality when compared with the general population of Americans. Protein intake among wheelchair users with MS was lower than the general population of Americans and constitutes a significant area of future interest given the importance of protein for preservation of muscle in persons with significant mobility limitations. To that end, a cogent next step may involve investigation of modest protein increase and the impact on function and muscle mass in wheelchair users with MS.

## Figures and Tables

**Table 1:** Participant demographic and clinical characteristics.

Variable	N = 128
**Age**, years ± SD	60.6 ± 9.7
**Sex**, *n* (%)	
Female	95 (74)
Male	33 (26)
**Marital Status**, *n* (%)	
Married	81 (63)
Single/Divorced/Separated/Widower	47 (37)
**Employment**, *n* (%)	
Yes	17 (13)
No	111 (87)
**Race**, *n* (%)	
White	114 (89)
Other	14 (11)
**Education**, *n* (%)	
Less than college degree	36 (28)
College degree or more	92 (72)
**MS Duration**, years ± SD	23.2 ± 9.5
**Type MS**, *n* (%)	
Relapsing Remitting	19 (15)
Progressive	109 (85)
**Wheelchair Type**, *n* (%)	
Power Wheelchair/Scooter	74 (58)
Manual Wheelchair	54 (42)

**Table 2:** Healthy eating index scores compared across diet questionnaire methods in 128 participants and the general population mean.

Healthy Eating Index Score 2015 (Maximum Score)	Mean ASA24	Mean DHQ III	Mean General Population [19]
**Total Score (100)**	60.9 *	66.2 *	58.7
**Adequacy Component Scores**			
Total Fruits (5)	3.0	4.0*	2.9
Whole Fruits (5)	3.3	4.2	4.2
Total Vegetables (5)	3.8	4.1 *	3.3
Greens and Beans (5)	3.4	3.9 *	3.1
Whole Grains (10)	3.9 *	3.3	3.0
Dairy (10)	5.5	5.1 *	6.0
Total Protein Foods (5)	4.5 *	4.5 *	5.0
Seafood and Plant Protein (5)	3.6 *	4.3	5.0
Fatty Acids (10)	5.1	5.4	4.1
**Moderation Component Scores**			
Refined Grains (10)	7.5	8.4	6.4
Sodium (10)	4.1	4.5 *	3.7
Added Sugar (10)	7.8 *	7.9 *	6.8
Saturated Fats (10)	5.3	6.6 *	5.1

Note. ASA24 = Automated Self-Administered 24-h Dietary Assessment Tool; DHQ III = Diet History Questionnaire III, * *p* ≤ 0.05.

**Table 3:** Bivariate correlations and follow-up paired sample t-tests among heathy eating index scores generated using the Automated Self-Administered 24-h Dietary Assessment Tool and the Diet History Questionnaire III.

Health Eating Index Score 2015	Correlation	Difference ± SD
**Total Score**	0.60 ***	5.33 ± 11.23 ***
**Adequacy Component Scores**		
Total Fruits	0.69 ***	0.95 ± 1.44 ***
Whole Fruits	0.62 ***	0.85 ± 1.62 ***
Total Vegetables	0.50 ***	0.28 ± 1.35 *
Greens and Beans	0.47 ***	0.52 ± 2.02 **
Whole Grains	0.43 ***	−0.63 ± 3.13 *
Dairy	0.54 ***	−0.33 ± 2.77
Total Protein Foods	0.23 **	0.02 ± 1.26
Seafood and Plant Protein	0.32 ***	0.69 ± 1.91 ***
Fatty Acids	0.26 **	0.26 ± 3.92
**Moderation Component Scores**		
Refined Grains	0.38 ***	0.45 ± 3.52
Sodium	0.46 ***	0.90 ± 3.03 **
Added Sugar	0.51 ***	1.25 ± 3.58 ***
Saturated Fats	0.33 ***	0.13 ± 2.60

Note. * *p* ≤ 0.05, ** *p* ≤ 0.01, *** *p* ≤ 0.001.

## Data Availability

The data presented in this study are available on request from the corresponding author. The data are not publicly available due to institutional guidelines.
